# Brightness perception under photopic conditions: experiments and modeling with contributions of *S*-cone and *ipRGC*

**DOI:** 10.1038/s41598-023-41084-7

**Published:** 2023-09-04

**Authors:** Tran Quoc Khanh, Peter Bodrogi, Babak Zandi, Trinh Quang Vinh

**Affiliations:** 1 Laboratory of Adaptive Lighting Systems and Visual Processing, Department of Electrical Engineering and Information Technology, Technical University of Darmstadt, 64289 Darmstadt, Germany; 2 ERCO GmbH, 58507 Lüdenscheid, Germany

**Keywords:** Psychology, Optics and photonics

## Abstract

In 1924, the CIE published and standardized the photopic luminous efficiency function. Based on the standardized curve, luminous flux in lumens, luminance in cd/m$^2$, and illuminance in lux are determined by an integral of the curve and the incident light spectra in photometers and are considered physical brightness. However, human brightness perception is not only weighted by this simple determination, but is a more complicated combination of all *L*-cones, *M*-cones, *S*-cones, rods and later *ipRGCs*, which was partly described by the equivalent brightness of Fotios et al. with the correction factor $(S/V)^{0.24}$. Recently, new research has demonstrated the role of *ipRGCs* in human light perception. However, it is still unclear how these signal components of the human visual system are involved in the overall human brightness perception. In this work, human brightness perception under photopic conditions was investigated by visual experiments with 28 subjects under 25 different light spectra. In this way, the contributions of the signal components can be investigated. An optimization process was then performed on the resulting database. The results show that not only the $L+M$ component, but also the *S*-cones and *ipRGC* play a role, although it is smaller. Thus, the visually scaled brightness model based on the database optimization was constructed using not only illuminance but also *S*-cones and *ipRGC* with $R^2$ of 0.9554 and *RMSE* of 4.7802. These results are much better than the brightness model after Fotios et al. using only *S*-cones ($R^2$ = 0.8161, *RMSE* = 9.7123) and the traditional model without *S*-cones and *ipRGC* ($R^2$ = 0.8121, *RMSE* = 9.8171).

## Introduction

The development of artificial light started at the beginning of the 20*th* century in the era of industrialization and electrification and was intended to provide indoor workplaces with light around the clock, thereby minimizing accidents at work. The research focus was initially based on the criteria of contrast perception, visual acuity, object size and adaptive luminance. From the 1960s, and then increasingly in the 1990s, the psychological component of lighting was intensively investigated with the aspects of homogeneity, luminance distribution, color rendering, color temperature, light direction with indirect and direct light components in the room^1–5^. For this integration of psychological components into lighting research, the chromatic aspects of the radiation components reaching the eye, e.g., the color fidelity, color discrimination, color difference^6,7^, were thus also considered.

Since the beginning of the 21*st* century, there have been three new focal points in lighting technology. The first focus—following the discovery of intrinsic photosensitive retina ganglion cells (ipRGCs)^8–14^—is the detection and quantification of the effects of optical radiation of different wavelengths and radiation components on sleep quality, work productivity, alertness, and well-being. The second focus is the research to describe the quality of the illumination of an object and of a scene in the room, substantially driven by the development of the LED-technology. Besides the color rendering index, further parameters of other properties such as color gamut, color memory and color saturation shall be added for the assessment of color quality^15–21^.

The investigations into the general brightness and, in special cases, spatial brightness as an important aspect of room illumination and scene perception, which form the subject of the present paper as the third focus, have been carried out experimentally and theoretically in vision science since the late 1960s^22–27^ and continued until today^28–32^ with an overall evaluation of the experimental methodology and the knowledge gained up to that time. There is a definition for spatial brightness formulated by IESNA (Illuminating Engineering Society of North America) and reproduced in^28^. Generally, spatial brightness describes the visual perception which is evoked by the incident light coming from a large part of the visual field being beyond the fovea area. This should be the case if the light user observes several objects in a room or on a street with different brightness levels.

For an accurate description and modeling of the characteristics of brightness, however, the following research questions are important for vision science:

- Is the parameter illuminance or luminance alone decisive for the perceived brightness of neutral (color tone-free) and colored objects?

If the answer to the above question is “*No*”, then:

- How can new numerical parameters for the mathematical modelling of perceived brightness be determined?

These questions are of high interest as numerous publications—when describing the test conditions and interpreting the test results—predominantly used only $V(\lambda )$—weighted parameters, e.g., the illuminance at the object level and the luminance of objects and of the surrounding walls of a room. Important for today’s vision science and lighting research for indoor applications, on the other hand, is also the knowledge of whether and to what extent—apart from $V(\lambda )$—there are other signals or signal combinations of the various photoreceptors of the retina, i.e. the cones (*L*, *M*, *S*), the rods and the intrinsically photosensitive ganglion cells (*ipRGCs*) that should contribute to the perception of brightness^25–28^ and thus to the perception of the overall atmosphere of an illuminated room and also, which wavelength ranges constitute the main part of electromagnetic radiation for perceived brightness.

**Figure 1:**
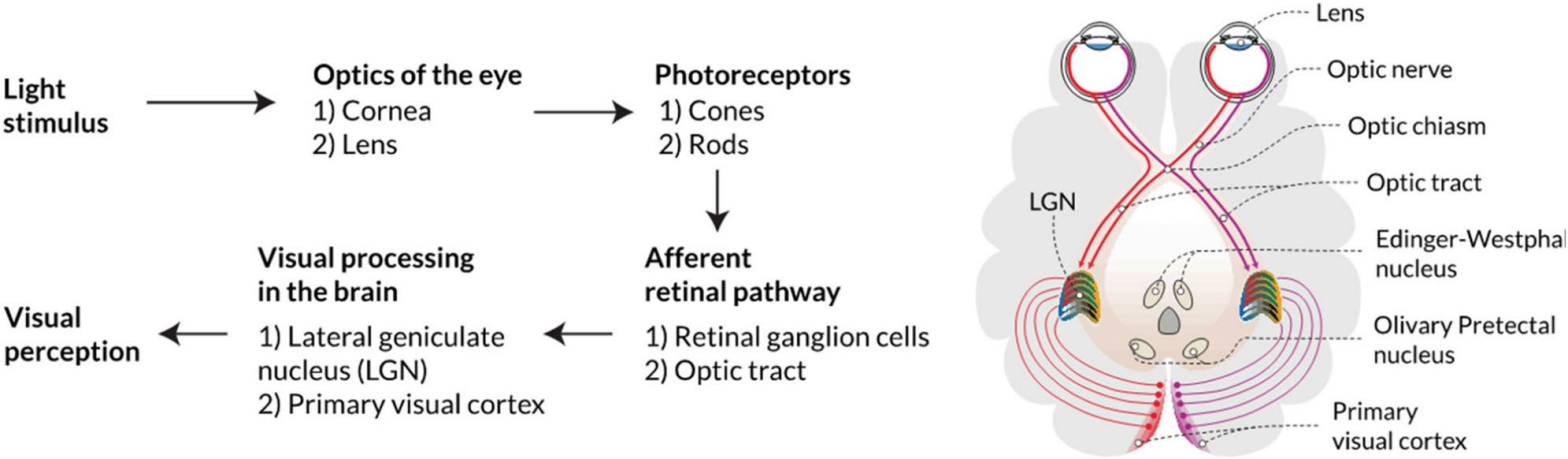
Simplified processing pathway of the human’s visual perception in response to light. The Figure is reprinted from (Zandi 2022,^35^) under CC BY—4.0 license.

From many visual experiences, it is known that white light with a higher blue light content, at a certain constant luminance or illuminance level, could evoke a higher brightness perception compared to warm white light^30,33^. The reason for this appearance is that not only the $V(\lambda )$-weighted signals (*L* + *M*), but also the signals of the other retinal mechanisms contribute to brightness perception. The rods responsible for night vision are irrelevant for indoor illumination situations at the daytime with a luminance at a higher level (higher than 10 cd/m$^2$)^27^. From the human eye physiological aspects, it is known, that electrical impulses are formed in the photoreceptors—after photon absorption—and transmitted to the ganglion cell layers (see Fig. 1). The parasol midget and small bistratified ganglion cells accounting for about 75% of all RGCs are responsible for transmitting frequency-coded action potentials to retinorecipient brain nuclei^34^. The ganglion cells’ axons project to the lateral geniculate nucleus (*LGN*) of the thalamus, which undertakes the gateway to the brain’s primary visual cortex $V_1$ in which visual sensory information is processed further (see Fig. 1).

The *LGN* consists of six different layers with the magnocellular (*M*) cells which are implemented in the lowest two pieces and the upper four layers contain the parvocellular (*P*) cells^36^. Between the *M*- and *P*-cells of the *LGN*, six additional layers are found containing the koniocellular (*K*) cells. The primary visual cortex received the encoded colour and brightness properties of visual stimuli from the parvocellular (*PC*), magnocellular (*MC*) and koniocellular (*KC*) pathways^37^ (see Fig. 1). The three different ganglion cell types and the corresponding cell layers in the *LGN* are responsible for the generation and processing of different visual information.

- The *P*- and *K*-cells in the *LGN* show opposing color responses. Midget ganglion cells provide cone opponent color information to the *PC*—pathway, derived from a subtraction of *L*- and *M*-cones (*L*–*M*, red–green opponency)^38^.
- Bi-stratified ganglion cells project the middle *KC*—layers and provide excitatory signals from *S*-cones with opposed inhibitory information from an additive *L*- and M-cones signal combination $(S - (L + M)$, blue–yellow opponency)^36^.
- Parasol ganglion cells project to the magnocellular (*M*) layer, delivering an achromatic luminance signal from *L*- and *M*-cones ($L + M$, luminance)^39^.

In the last five decades, research activities were performed concerning brightness experiments with colored and conventional white light and modeling^27,40–42^, which leaded to a summarizing paper of the CIE (International Commission on Illumination) in^43^. All the models included in this fundamental paper considered the contributions of the opponent channels (*L*–*M*) and $(S - (L + M)$) indirectly by implementing the chromaticity x and y into a function with the luminance from the achromatic signal $(L + M)$. In a doctoral thesis on the photopic brightness in indoor lighting, Pepler^33^ varied the polychromatic white light source spectra and the luminance on a homogeneous and diffusely reflecting wall in a real room without daylight incidence and found, that under the defined test conditions with white lights, the most consistent model corresponding to the subjective evaluations of the test persons is a simple model of Fotios et al. from 1998^26^ in which the so-called equivalent luminance ($L_{eq}$) can be defined according to Eq. (1).

1 $$\begin{aligned} L_{eq} = L_{v} (S/V)^{0.24} \end{aligned}$$

**Figure 2:**
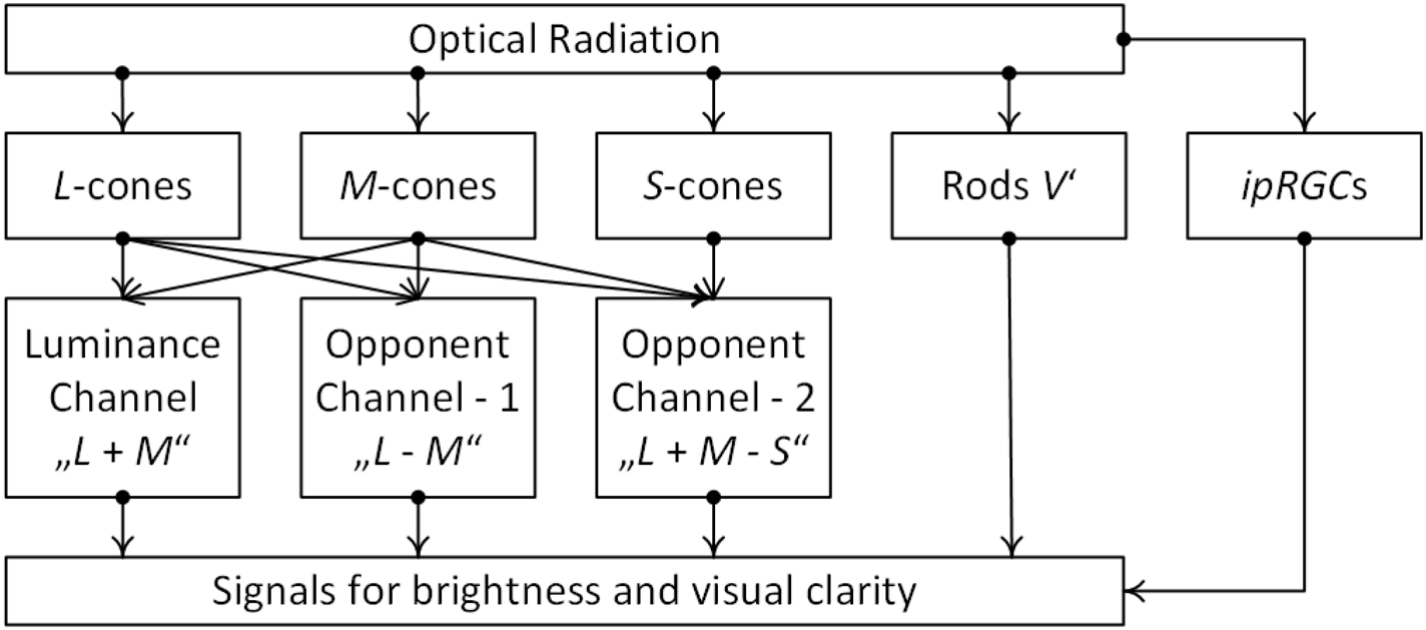
Hypothesis for signal components of the human visual system to form a numerical parameter for brightness perception. Image source: Laboratory of Adaptive Lighting Systems and Visual Processing, Technical University of Darmstadt.

**Figure 3:**
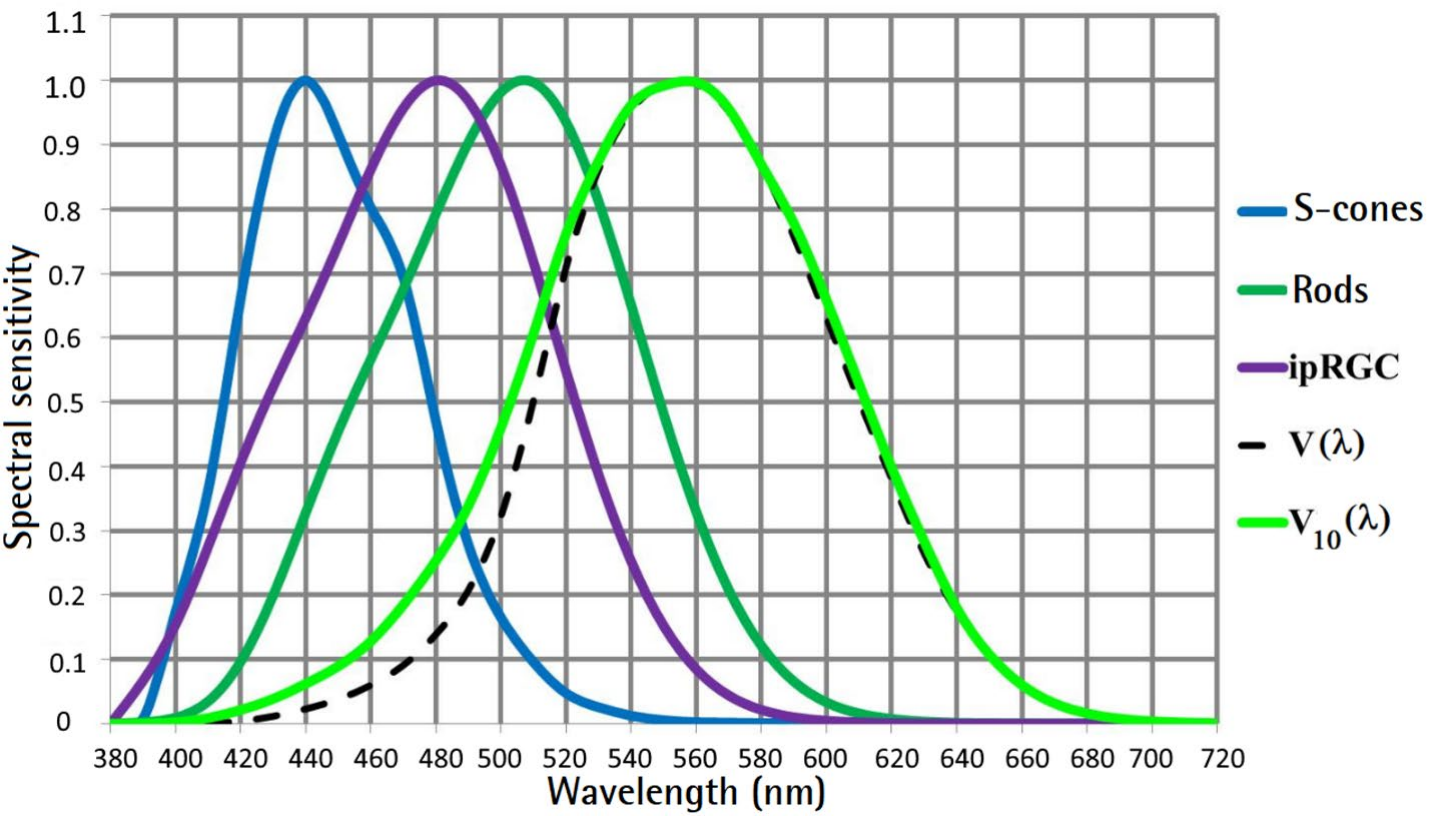
Spectral sensitivity of rods, *S*-cones and photosensitive ganglion cells (*ipRGCs*) compared to the $V(\lambda )$ function and the $V_{10}(\lambda )$ function, Image source: Laboratory of Adaptive Lighting Systems and Visual Processing, TU Darmstadt.

In Eq. (1), the exponent of the $V(\lambda )$-weighted luminance ($L_v$) equals 1. This means that the photopic luminance does not experience signal compression. To calculate the signals *S* or *V*, the relative spectral radiant flux of the light source must be multiplied by the spectral sensitivity function of the *S*-cones or by the $V(\lambda )$ function, respectively, and this product must be integrated in the visible wavelength range. It should be emphasized that, in accordance with Eq. (1)—*S*-cones (see Fig. 2) should contribute to the formation of the signals for the attribute luminance (at least in the photopic region, in which the rod signals are not available, see Figs. 2 and 3). With the discovery of the new type of ganglion cells, the *ipRGCs*, some research studies have been performed aiming to answer the question if also the *ipRGC*-signals could contribute to the brightness perception in the photopic vision range. According to the latest studies on the field of neurophysiology, there are some reasons to assume that *ipRGCs* interact in at least two different ways with the visual channels^44^. In one way, the so-called $M_4$- subtype *ipRGCs* project to the *LGN* and might contribute to the human’s brightness perception^45^. In another way, a group of $M_1$-subtype *ipRGCs* builds signal connections with upstream dopaminergic amacrine cells and possibly influencing the light adaptation state^46^. In this context, *ipRGCs* can influence other *RGC* types and the communication between cones and related bipolar cells. The studies of Zele et al.^44^ in 2018 and Yamakawa et al.^47^ in 2019 had found several *ipRGC*-signals in the brightness perception. These findings in the laboratory conditions should be validated due to their small sample size and number of visual stimuli.

In the present work, the attribute brightness is modelled with the *S*-cone signals and the *ipRGC* signals based on the results from an empirical study, which was comprehensively conducted in a real room using a higher number of test persons and visual stimuli (luminance of the objects and spectra of the light sources). This work’s key outcome is an empirical-based brightness perception model that includes the *S*-cone and the *ipRGC* contribution, with which the human’s brightness sensation can be predicted more accurately compared to the *V*($\lambda$)-based counterparts like the luminance.

## Experimental method of the subjective study

**Figure 4:**
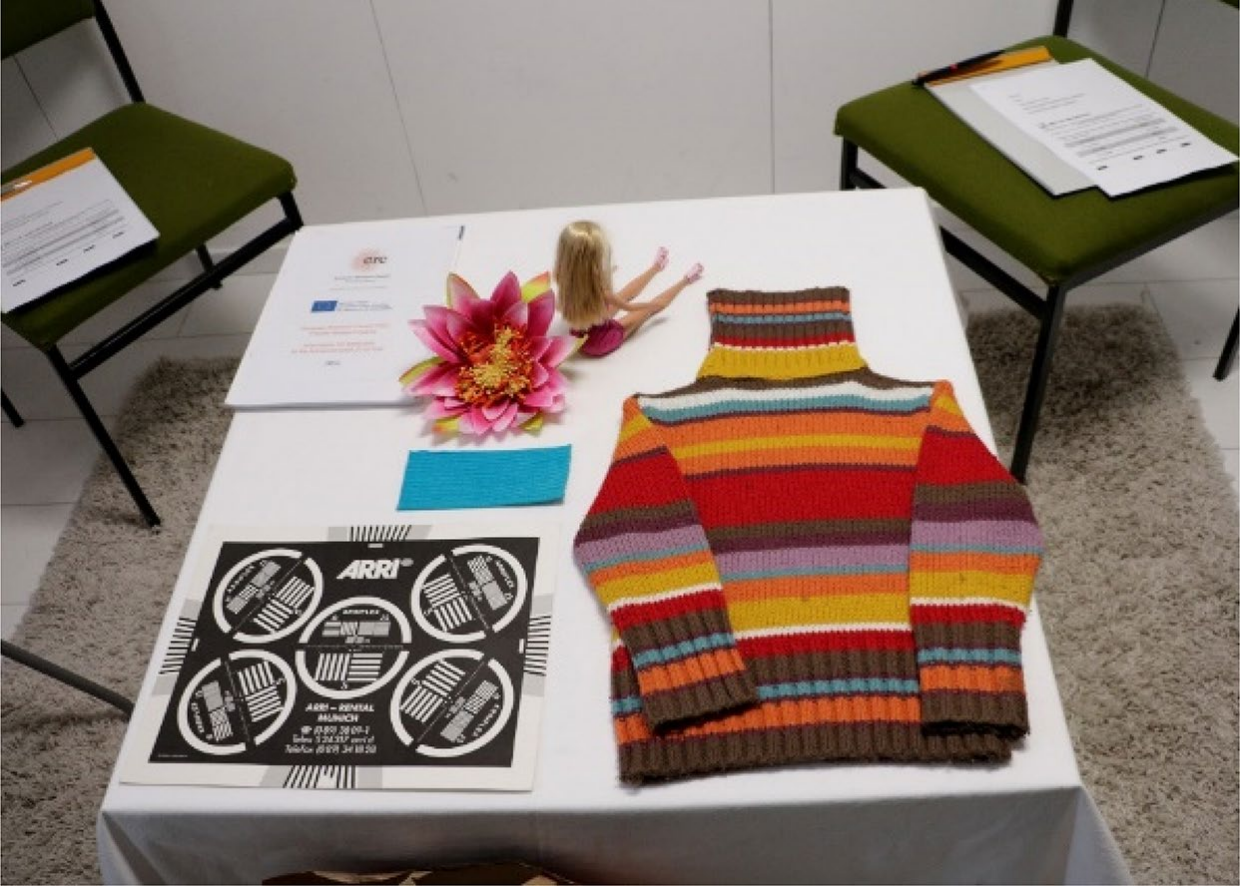
Experimental set-up for the subjective assessment of brightness.

The conducted study took place in a real, office-like room without daylight, with homogeneous white and matt painted walls and neutral flooring. The subjects sat on chairs and looked at a homogeneously illuminated table with a white tablecloth (see Fig. 4). The subjects first adapted to the white tablecloth for about 2 min according to the research result of Fairchild et al.^48^ on the time course of the chromatic adaptation during colour-appearance judgment. The subjects scaled their impression of the brightness of a scene with various 3-dimensional coloured and achromatic objects (doll with long hair, jumper with colourful patterns, artificial water lily, black and white test pattern with fine line structure), which were illuminated in this room with two *RGBW* LED lights with light diffusing covers causing a uniform and diffuse illumination on the objects.

**Figure 5:**
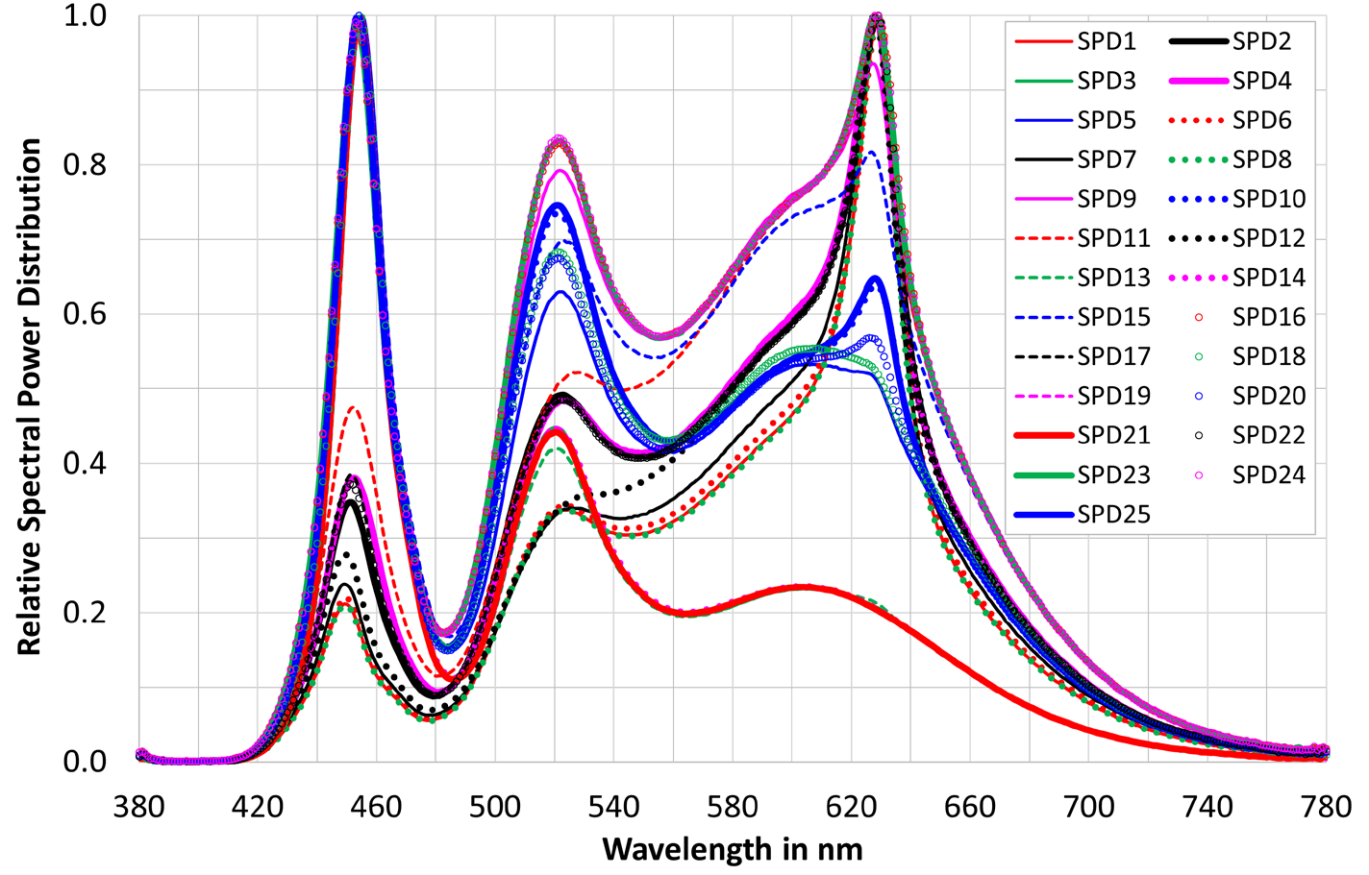
Illumination spectra in the 25 lighting situations examined in the subjective study for brightness (Data source: Laboratory of Adaptive Lighting Systems and Visual Processing, Technical University of Darmstadt.

The horizontal illuminance on the table was 45 lx, 90 lx, 470 lx, 1000 lx and 2000 lx respectively, so that the vision condition is photopic. For the condition of 45 lx, the luminance on the table has been 13.1 cd/m$^2$. The correlated colour temperature (*CCT*)—at each illuminance level—was also varied: 2700 K, 3100 K, 4100 K, 5000 K and 10,000 K and covered a range of colour temperatures in private and professional rooms in the evening and daytime. Thus, the subjects were shown 25 different ($E_v\,x\,CCT$) combinations, waiting about 1.5 min for re-adaptation after adjusting each light combination until the subjective evaluation was made. The illuminating RGBW LED light sources were optimised to ensure high colour rendering index levels (89 $\le$ IES TM30-20 $R_f \le$ 93) for each spectrum (5 $\times$ 5 = 25). The illumination spectra are shown in Fig. 5. Table 1 shows the colorimetric and photometric parameters of the 25 spectra which have been presented to the test persons in a randomized order. Table 2 lists the $\alpha$-opic illuminances (*L*-cone-opic, *M*-cone-opic, *S*-cone-opic, rod-opic and melanopic equivalent daylight D65 illuminance values) according to CIE S 026/E: 2018)^14,49^. Twenty-eight subjects with normal or corrected visual acuity and without colour vision deficiencies were recruited from a pool of students at the University. After they arrived in the lighting laboratory, they were tested with “*The Standard Pseudoisochromatic Plates for Acquired Color Vision Defects*”^50^. This study was approved by the ethics committee of the Technical University of Darmstadt and carried out following the ethical principles of the Declaration of Helsinki. All subjects were informed about the content of the study. Signed consent was obtained from the participants before the experiment took place.

**Table 1:** Photometric and colorimetric data of the illumination spectra (SPD) used in the experiment.

Spectrum/parameters	*CCT* in K	*Duv*	$E_v$ in lux	*x*	*y*	IES TM30-20 $R_f$	IES TM30-20 $R_g$
$SPD_1$	2693	$8.68 \cdot 10^{-4}$	1000	0.4619	0.4134	92	104
$SPD_2$	3096	$2.87 \cdot 10^{-3}$	470	0.4343	0.4103	93	103
$SPD_3$	10,021	$4.43 \cdot 10^{-3}$	90	0.2778	0.2935	89	101
$SPD_4$	3107	$9.83 \cdot 10^{-4}$	90	0.4308	0.4043	93	103
$SPD_5$	5008	$4.42 \cdot 10^{-4}$	45	0.3450	0.3524	91	102
$SPD_6$	2705	$8.28 \cdot 10^{-4}$	2000	0.4609	0.4131	92	104
$SPD_7$	2693	$-5.24 \cdot 10^{-5}$	90	0.4604	0.4106	91	104
$SPD_8$	2691	$8.87 \cdot 10^{-4}$	470	0.4621	0.4135	92	104
$SPD_9$	4100	$-2.84 \cdot 10^{-5}$	90	0.3761	0.3740	92	102
$SPD_{10}$	5008	$3.89 \cdot 10^{-3}$	2000	0.3456	0.3599	92	104
$SPD_{11}$	3104	$-2.79 \cdot 10^-4$	45	0.4294	0.4007	90	102
$SPD_{12}$	2698	$-4.11 \cdot 10^{-4}$	45	0.4593	0.4094	89	103
$SPD_{13}$	10,029	$1.32 \cdot 10^{-3}$	45	0.2797	0.2897	89	101
$SPD_{14}$	10,012	$3.57 \cdot 10^{-3}$	2000	0.2784	0.2925	89	101
$SPD_{15}$	4097	$-2.70 \cdot 10^{-3}$	45	0.3746	0.3674	90	101
$SPD_{16}$	4092	$9.11 \cdot 10^{-4}$	2000	0.3771	0.3766	92	103
$SPD_{17}$	3103	$1.15 \cdot 10^{-3}$	2000	0.4314	0.4049	93	104
$SPD_{18}$	5011	$2.99 \cdot 10^{-3}$	90	0.3454	0.3579	92	102
$SPD_{19}$	10,003	$3.99 \cdot 10^{-3}$	1000	0.2782	0.2931	89	101
$SPD_{20}$	5006	$1.50 \cdot 10^{-3}$	470	0.3452	0.3547	92	103
$SPD_{21}$	10,004	$3.26 \cdot 10^{-3}$	470	0.2787	0.2922	89	101
$SPD_{22}$	3099	$1.68 \cdot 10^{-3}$	1000	0.4324	0.4066	93	103
$SPD_{23}$	4101	$1.10 \cdot 10^{-3}$	470	0.3768	0.3768	92	103
$SPD_{24}$	4109	$1.10 \cdot 10^{-3}$	1000	0.3765	0.3766	92	103
$SPD_{25}$	5007	$4.36 \cdot 10^{-3}$	1000	0.3458	0.3609	92	104

**Table 2:** $\alpha$-Opic illuminance values of the 25 illumination spectra (SPD) in the experiment (L-cone-opic, *M*-cone-opic, *S*-cone-opic, rod-opic and melanopic equivalent daylight D65 illuminance (is denoted as—$DI$) according to CIE S 26/E: 2018)^14,49^.

Spectrum/parameters	$E_v$ in lx	ME-DI	Rh-DI	Lc-DI	Mc-DI	Sc-DI
$SPD_1$	1000	431	528	1018	784	265
$SPD_2$	470	234	278	474	386	157
$SPD_3$	90	104	102	89	94	120
$SPD_4$	90	45	54	91	74	33
$SPD_5$	45	36	38	45	42	35
$SPD_6$	2000	863	1055	2035	1569	537
$SPD_7$	90	38	47	92	70	25
$SPD_8$	470	203	248	479	368	124
$SPD_9$	90	61	67	90	80	54
$SPD_{10}$	2000	1581	1691	1985	1875	1473
$SPD_{11}$	45	22	26	45	37	17
$SPD_{12}$	45	19	23	46	35	13
$SPD_{13}$	45	52	51	45	47	61
$SPD_{14}$	2000	2290	2261	1983	2088	2671
$SPD_{15}$	45	31	33	45	40	28
$SPD_{16}$	2000	1348	1486	1997	1783	1175
$SPD_{17}$	2000	1010	1190	2018	1645	718
$SPD_{18}$	90	71	75	89	84	67
$SPD_{19}$	1000	1146	1132	991	1045	1332
$SPD_{20}$	470	372	396	467	439	359
$SPD_{21}$	470	539	532	466	491	629
$SPD_{22}$	1000	502	594	1009	822	351
$SPD_{23}$	470	318	350	469	419	276
$SPD_{24}$	1000	677	746	999	893	589
$SPD_{25}$	1000	791	847	992	938	731

**Figure 6:**
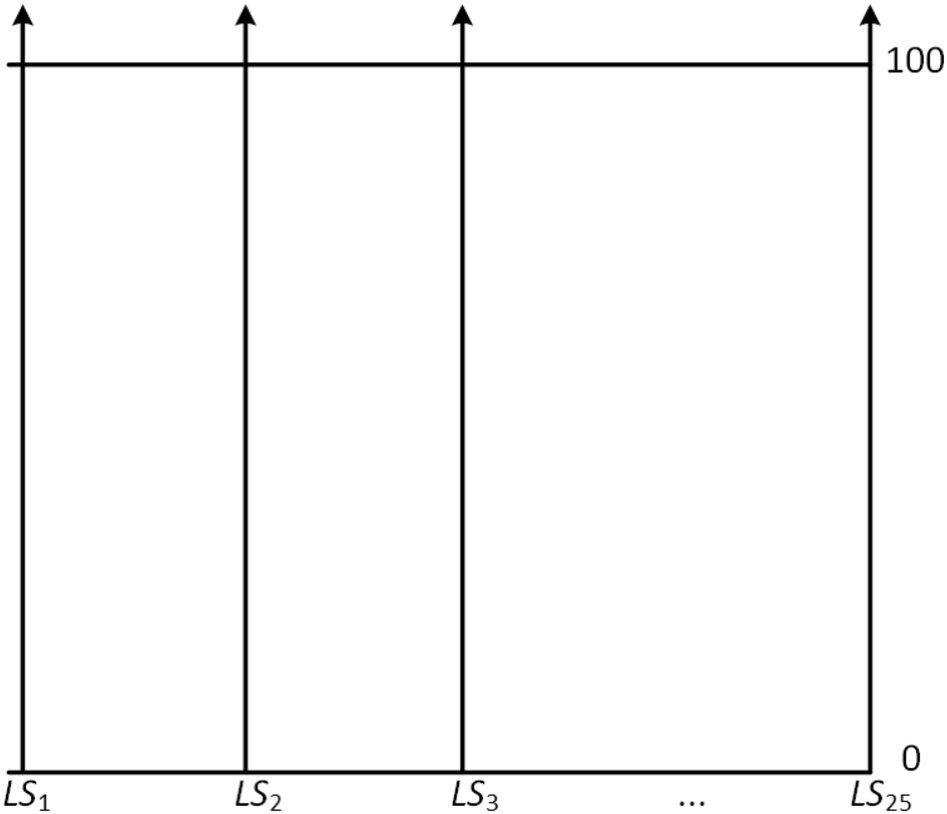
Rating scale for the subjective assessment of brightness perception ($LS_i$, i = 1–25)) with the anchor points shown on the right: 0 and 100)^51^.

Before the main experiment, the test leader explained the procedure of the experiment with questionnaires in about 10 min in which the test person adapted to the lighting condition in the test room. Subsequently, a training was conducted with different light settings and the same questionnaire which contains a continuous brightness scale between 0 and 100 (see Fig. 6). In this training phase of about 10 min, 2 extreme light settings such as very bright, very dark and 3 settings in between were also presented to create so-called anchor stimuli. The training results were not included in the final evaluation of the results.

For a better understanding of the brightness test, it is beneficial to describe the instructions given by the experimenter to the subjects (see also Fig. 6):

**Brightness**: “The term brightness is used in an everyday sense in that you evaluate the whole table with the tablecloth—compared to the reference situation—according to brightness. How bright does the table appear compared to the reference situation? The reference situation is shown several times in the training phase and corresponds to a brightness value of 100. Complete darkness corresponds to a value of 0. Please evaluate your impression of brightness (*H*) by looking at the table, i.e., the white tablecloth. In the training phase, you can memorise your brightness impression of the reference scene with H=100, which is shown repeatedly. You can tick the scale—according to your brightness impression. After each adjustment of a new light source, please look at the table for 90s first. After this adaptation, you can then evaluate the brightness. Please do not look at your own hand, nor at the faces of the other persons, only at the table, i.e., the white tablecloth. Please first decide in which third the current situation is (top, middle or bottom) and then tick the scale—corresponding to your impression of brightness—within this third”. The referent situation is the light SPD14 in Tables 1 and 2.

In the main experiment, the 25 spectra mentioned above were presented randomly. Between each setting in the main experiment, 1.5 min for perception and one additional minute for evaluation were planned. The total experimental duration was therefore about 80–85 min long. The test persons had been paid for their test efforts according to the regulation of the University.

## Modelling brightness

The subjects’ mean visual scale scores were modeled for brightness and using the quantity M, defined in Eq. (2).

2 $$\begin{aligned} M = a \cdot [E_v^\gamma (\alpha \cdot S^\delta + \beta \cdot G^\delta )] + b \end{aligned}$$

with

3 $$\begin{aligned}{} & {} E_v=683 \int _{380}^{780}E_{\lambda ,abs.} (\lambda ) \cdot V_{\lambda } (\lambda ) \cdot d\lambda \end{aligned}$$

4 $$\begin{aligned}{} & {} E_{\lambda ,rel.}(\lambda )=\dfrac{E_{\lambda ,abs.} (\lambda ) \cdot 100}{\int _{380}^{780}E_{\lambda ,abs.} (\lambda ) \cdot V_{\lambda } (\lambda ) \cdot d\lambda } \end{aligned}$$

5 $$\begin{aligned}{} & {} S=\frac{\int _{380}^{780}E_{\lambda ,rel.} (\lambda ) \cdot S_{\lambda } (\lambda ) \cdot d\lambda }{\int _{380}^{780}E_{\lambda ,rel.} (\lambda ) \cdot V_{\lambda } (\lambda ) \cdot d\lambda } \end{aligned}$$

6 $$\begin{aligned}{} & {} G(\text {that denotes for ipRGC})=\frac{\int _{380}^{780}E_{\lambda ,rel.} (\lambda ) \cdot ipRGC_{\lambda } (\lambda ) \cdot d\lambda }{\int _{380}^{780}E_{\lambda ,rel.} (\lambda ) \cdot V_{\lambda } (\lambda ) \cdot d\lambda } \end{aligned}$$

$S(\lambda )$, $V(\lambda )$, $ipRGC(\lambda )$ are shown in Fig. 3. $E_{\lambda ,abs.}(\lambda )$ is the absolute spectral irradiance distribution on the object plane in W/(nm m$^2$) and $E_{\lambda ,rel.}(\lambda )$ is the relative spectral distribution derived from $E_{\lambda ,abs.}(\lambda )$.

The symbols in Eq. (2) have the following meaning.

- The parameter $\gamma$ is an exponent for the compression of the illuminance and the parameter $\delta$ is an exponent for the compression of the *S* and the *G* signals (*G* denotes in this paper the *ipRGC* signal).
- The parameters *a* and *b* are the parameters of a linear transformation to fit the calculated $M_i$ values to the mean visual scale values $VSB_i$ (for brightness) of the test subjects for the 25 light source spectra (*i* = 1–25, see Fig. 5) with the smallest error *RMSE*. The error size root mean square error (*RMSE*) is defined accordingly in Eq. (7).

7 $$\begin{aligned} RMSE=\sqrt{\frac{\Sigma _{i=1}^{100} {(M_i-VSB_i)}^2}{25}} \end{aligned}$$

The parameters $\alpha$ and $\beta$ of Eq. (2) are weighting parameters of the relative signals of the mechanisms *S* (*S*-cones) and *G*(*ipRGCs*: intrinsically photosensitive retinal ganglion cells). The relative signals were calculated from the 25 relative spectra. To calculate these signals (*S* and *G*), the relative spectral radiant flux of the light source must be multiplied by the spectral sensitivity function of the *S*-cones or the *ipRGCs* (published in the CIE-publication^14^ and this product shall be integrated in the visible wavelength range. Afterwards, these signals are divided by the so-called *V*-signal. To calculate the *V*-signal, the relative spectral radiant flux of the light source shall be multiplied by the $V(\lambda$)-function and this product shall be integrated in the visible wavelength range.

**Table 3:** Parameter values of Eq. (2) and the corresponding values of the error (*RMSE*) of Eq. (7) for brightness. Weight of S-cones: $$\alpha $$; weight of ipRGC: $$\beta $$; exponent for illuminance: $$\gamma $$; exponent for S and G signals: $$\delta _1$$ and $$\delta _2$$, respectively; *a*, *b*: parameters of the linear transformation to approximate the mean visual scale values of the subjective study; *Optimization in row no. $$1^*$$: these parameter values represent Eq. (1) according to Fotios and Levermore^26^, where $$\alpha , \beta , \gamma $$ and $$\delta $$ were constant and only *a*, *b* were optimized; **Optimization in row no. $$18^{**} $$: it is in the traditional form without S and G=ipRGC, where $$\alpha $$ was 1 and only *a*, *b* were optimized; Source: Technical University of Darmstadt.

No.	Model parameters–synthesis							Model quality parameter			
	$\alpha (S)$	$\beta (G)$	*a*	*b*	$\gamma (E_v)$	$\delta _1(S)$	$\delta _2(G)$	*RMSE*	$R^2$	$a_{\text {Fit-quality}}$	$b_{\text {Fit-quality}}$
Model with S after Fotios: $M=a \cdot [E_v \cdot (S/V)^{0.24} + b$ defined for $\alpha =1$, $\beta =0$, $\gamma =1$ & $\delta =0.24$											
1$^*$	1	0	0.0362	40.9464	1	0.24	0.24	9.7123	0.8161	1	-2$\cdot 10^{-10}$
Model with S and/or G=ipRGC: $M=a \cdot [E_v^\gamma (\alpha \cdot S^{\delta _1} + \beta \cdot G^{\delta _2})] + b$ investigated parameters ($\alpha$, $\beta$, $\gamma$, $\delta _1$ & $\delta _2$)											
2	1	0	3.5298	20.0113	0.417	0.116	0.116	5.5768	0.9394	1	5$\cdot 10^{-10}$
3	0.8	0.2	3.4912	20.0069	0.417	0.116	0.15	5.5707	0.9395	1	2$\cdot 10^{-5}$
4	0.67	0.33	3.7084	19.4514	0.41	0.132	0.132	5.5536	0.9399	1	3$\cdot 10^{-7}$
5	0.6	0.4	3.4534	20.0033	0.417	0.116	0.15	5.5655	0.9396	1	2$\cdot 10^{-9}$
6	0.5	0.5	5.2903	15.1363	0.367	0.1200	0.1200	5.2872	0.9455	1	-3$\cdot 10^{-9}$
7	0.4	0.6	3.4164	20.0003	0.417	0.1160	0.1500	5.5609	0.9397	1	1$\cdot 10^{-9}$
8	0.33	0.67	2.3649	23.4100	0.461	0.1500	0.1500	5.8370	0.9336	1	6$\cdot 10^{-9}$
9	0.2	0.8	3.380	19.9979	0.417	0.1160	0.1500	5.5570	0.9398	1	3$\cdot 10^{-9}$
10	0	1	2.6766	22.2293	0.444	0.1710	0.1710	5.7458	0.9356	1	1$\cdot 10^{-11}$
11	0.92	0.92	2.8746	15.1363	0.367	0.1200	0.1200	5.2872	0.9455	1	3$\cdot 10^{-11}$
12	0.92	0.92	2.8562	15.0650	0.367	0.1100	0.1100	5.2610	0.9460	1	4$\cdot 10^{-8}$
13	0.92	0.92	2.8372	15.0042	0.367	0.1000	0.1000	5.2457	0.9463	1	1$\cdot 10^{-7}$
14	0.92	0.92	2.8075	14.9332	0.367	0.0850	0.0850	5.2434	0.9464	1	1$\cdot 10^{-8}$
15	0.92	0.92	2.7765	14.8867	0.367	0.0700	0.0700	5.2661	0.9459	1	7$\cdot 10^{-9}$
16	0.92	0.92	2.6321	14.9630	0.367	0.0070	0.0070	5.6243	0.9383	1	1$\cdot 10^{-8}$
17	1	0.5	8.9974	− 1.3307	0.2629	0.0740	0.0424	4.7802	0.9554	1	1$\cdot 10^{-7}$
Model without contribution of *S* and $G=ipRGC$: $M = a \cdot E_v^\gamma + b$											
18$$^{**}$$	–	–	0.028	40.641	1	–	–	9.8171	0.8121	1	1$$\cdot 10^{-9}$$
Ideal fit-quality: visual scaled brightness $(VSB)= 1 \cdot M + 0$ $\rightarrow$ $a_{\text {Fit-quality}}=1$; $b_{\text {Fit-Quality}}=0$ and $R^2=1$; $RMSE=0$											

The calculation and optimisation of the model parameters according to Eq. (2) has several steps (see Table 3):

Step 1: Based on Eq. (1) of Fotios, the model parameters are suggested as $$\alpha =1$$, $$\beta =0$$, $$\gamma =1$$ & $$\delta _1=\delta _2=\delta =0.24$$ when the brightness weighted by $$V(\lambda )$$ is not considered good and should be replaced by an equivalent luminance. The quality of this model without ipRGC (according to Fotios et al.^26^ listed in row $$1^*$$ of Table 3) as well as the model without contributions of ipRGC- and S-channels (listed in row $$18^{**}$$) has a quality with *RMSE* of about 9.7–9.8 and with the correlation coefficient $$R^2$$ of about 0.81–0.82.

Step 2: In rows 2–11, the process of modeling starts by varying $\alpha$ and $\beta$ in the 1 range so that a and b follow for a optimal result. Sometimes $\gamma$ and $\delta _1$ are also changed in the optimization trials. The result is a correlation coefficient $R^2$ in the range of about 0.94 and *RMSE* of about 5.5–5.84. The best case in this series occurs in the row 6 with $R^2$ of 0.9455 and *RMSE* of 5.287.

The values of the row 6 differ from those of the row 11 if the pair ($\alpha$; $\beta$) is varied from (0.5; 0.5) to (0.92; 0.92) by keeping the values of $\gamma$ and $\delta$ constant. With the new values of the parameters *a* and *b* in the row 11, the optimization results of the row 11 are equal those of the row 6.

Step 3: In the next step, from row 12 to row 16, the values of the parameters ($\alpha$; $\beta$) and $\gamma$ are kept constant and the parameter $\gamma$ together with the value pair (*a*, *b*) are optimized so that the *RMSE* is minimized and the $R^2$ is maximized. The best results are found at a value of $\delta$ being about 0.0085.

Step 4 (final step): A global optimization is performed with varying all parameters ($\alpha$, $\beta$, $\gamma$, $\delta _1$ for S, $\delta _2$ for G) and correlated parameters (*a*, *b*). The result in row 17 shows that it is the best case with $R^2$ of 0.9554 and *RMSE* below 5 (4.7802). The results give a lot of valuable information:

- Weighting factors $\alpha$ and $\beta$ are not equal for S- and ipRGC-signal.
- The exponent $\delta _2$ for compressing the output of ipRGC is much lower than that of $\delta _1$ of the S-cone-signal.
- The exponents $\delta _1$ and $\delta _2$ are much lower than the compression exponent $\gamma$ of the $E_v$-signal.
- The exponent $\gamma$ of $E_v$ is not 1.0 (compression) and lower at 0.2629.

Table 3 shows that the smallest error ($RMSE = 4.7802$) results in the row no. 17, where the weighting of the *S*-signal is equal to 1.0 and the weighting of the *ipRGC*- (*G*-) signal is equal to 0.5. If the parameters ($\alpha$, $\beta$) are being optimized, then the same result is obtained: i.e., there is a global optimum at $\alpha = 1.0$, $\beta = 0.5$ with $RMSE=4.78$, $R^2=0.955$, $a_{\text {Fit-Quality}} = 1$ and $b_{\text {Fit-Quality}} = 1 \cdot 10^{-7}$. For other fixed ($\alpha$, $\beta$) parameter values (other rows in Table 3) there are only slightly larger errors (*RMSE* higher 5) and only slightly different optimum ($\gamma$, $\delta$) values. This analysis indicates that, based on the present experimental data set with 28 test persons and with 25 different absolute light source spectra, it is not possible to decide which signal (*S* or $G = ipRGC$ or both) is decisive for brightness perception.

For modeling perceived brightness, the row no. 17 of Table 3 with the smallest error *RMSE* is proposed here in the Eq. 8 which is scaled so that the model value *M* reflects the same scale used by the subjects.

8 $$\begin{aligned} M_{\text {Eq.8}}=8.9974 \cdot [E_v^{0.2629} \cdot (1.0 \cdot S^{0.074} + 0.5 \cdot G^{0.0424})]-1.3307 \end{aligned}$$

**Figure 7:**
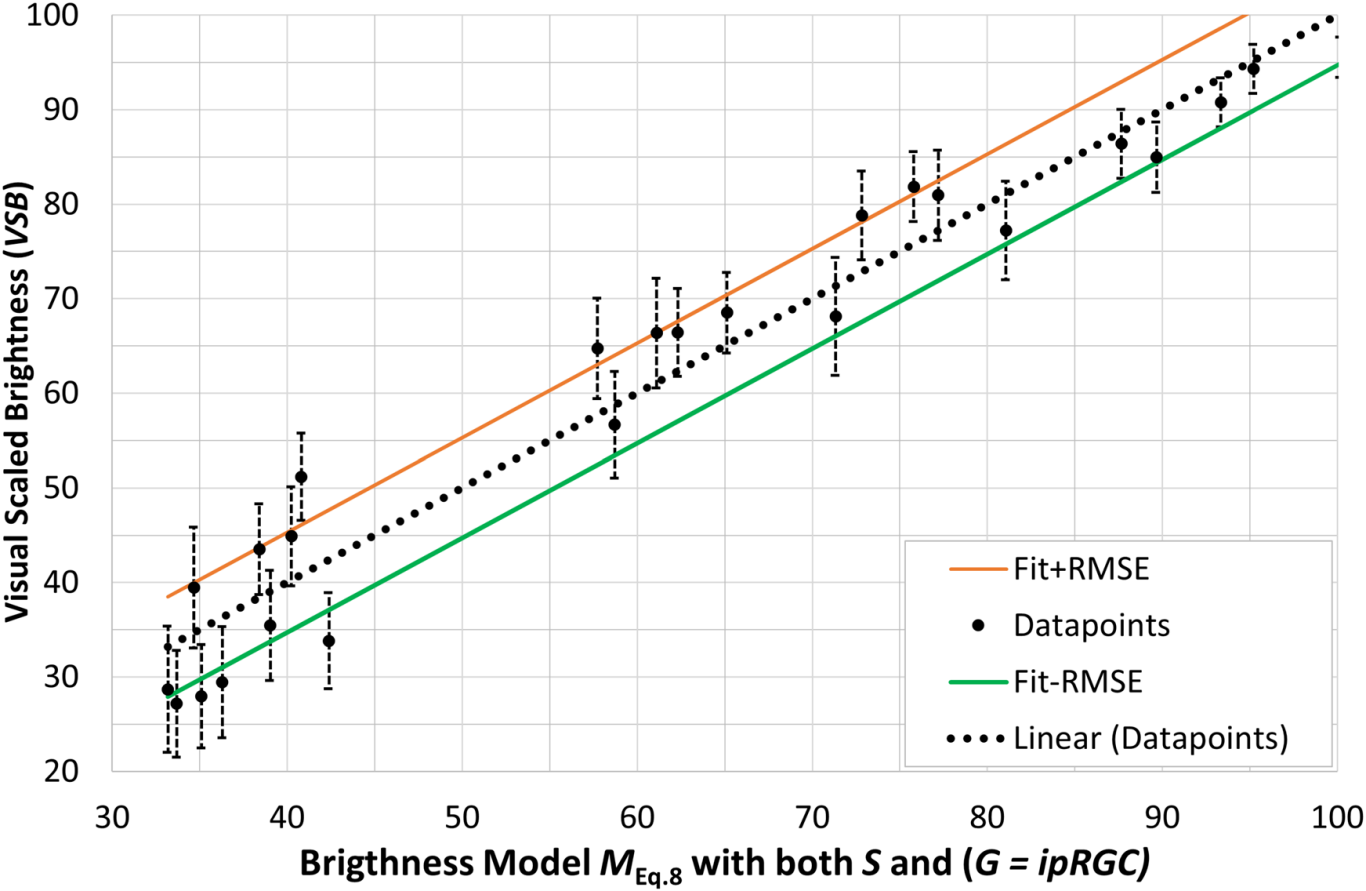
Mean visually scaled brightness values (*VSB*) of all observers from the experiment in Section “Experimental method of the subjective study” and their 95% confidence intervals as a function of the parameter $M(\text {brightness})$ of Eq. (8) with $R^2=0.955$, $RMSE= 4.78$.

Figure 7 visualizes the mean, visually scaled brightness values of all observers from the experiment in Section “Experimental method of the subjective study” and their 95% confidence intervals as a function of the parameter $M(\text {brightness})$ of Eq. (8), with $R^2=0.9554$ and $RMSE = 4.7802$.

**Figure 8:**
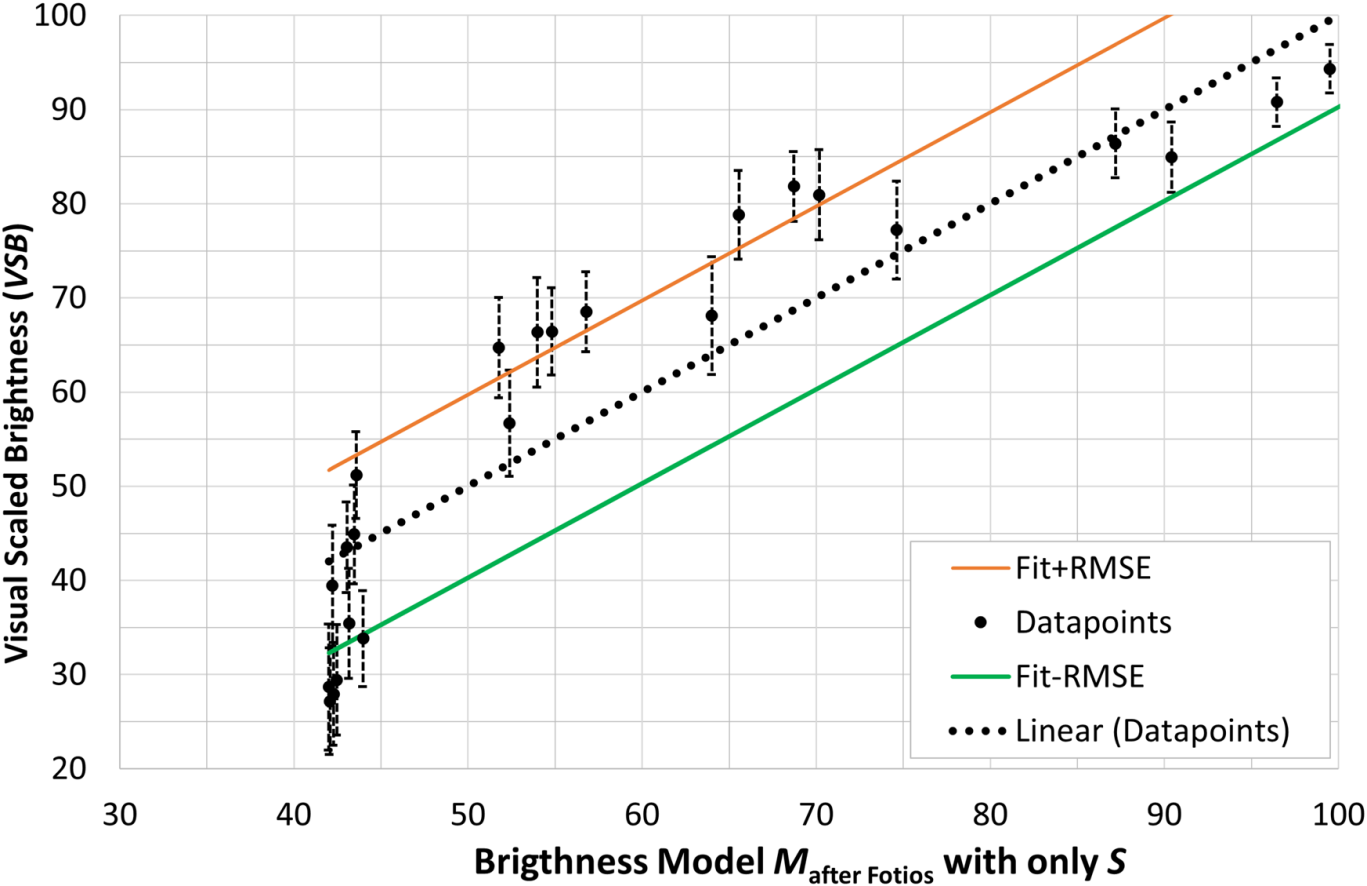
Mean visually scaled brightness values (*VSB*) of all observers from the experiment in Section “Experimental method of the subjective study” and their 95% confidence intervals as a function of the parameter $M(\text {brightness})$ according to Fotios et al.^26^, $M_{\text {after Fotios}} = 0.036 \cdot [E_v \cdot (S/V)^{0.24}] + 40.946$ with $R^2=0.8161$ and $RMSE=9.7123$.

As mentioned above, the parameters $\alpha$, $\beta$, $\gamma$ and $\delta$ in row no. $1^*$ of Table 3 remained constant and only the parameters (*a*, *b*) were optimized, which linearly fit the scale of the Fotios-Levermore model^26^ to the brightness scale of the present experiment. In this case, a significantly larger error results, $RMSE = 9.71$ (see Fig. 8). The possible reason for this is that the Fotios–Levermore model does not compress the illuminance scale ($E_v$), i.e., works with exponent $\gamma =1.0$.

From the comparison of Figs. 7 and 8 it is evident how important it is, on the one hand, to compress the scale of the illuminance (or luminance) with a suitable exponent and, on the other hand, to refine the model with the implementation of “*blue-sensitive*” signals that represent both components, *S*-cones and *ipRGCs*.

## Discussion and summary

If the signals of the two blue-light-containing channels *S*-cone and *ipRGC* (or one of them, e.g., the *S*-signal) are considered as in Eq. (8), we obtain a better prediction of the mean visually scaled results of lightness perception. According to the results of the present study, either the *ipRGC* signal (here also called the *G* signal) or the *S* signal or both signals play an important role. The question of whether the *S*- or the *G*-signal is critical could not be answered from the results of the present study.

In any case, it is very important to include a “*blue-sensitive*” signal in the model. An implementation of a compression of the scales of the input variables ($E_v$, *S*, *G*) by suitable exponents $(<1)$, such as $\gamma$ and $\delta$ in Eq. (2), is also relevant because signal compression is generally a significant property of the human perceptual system.

The attention to a “*blue-sensitive*” signal (*S* or *G* = *ipRGC*) means for vision science, lighting technology and lighting designers that the range around 420–520 nm (see the blue and purple curves in Fig. 3) plays an important role in the design of light spectra and the brightness perception of a scene in a room.

## Data Availability

The datasets used and/or analysed during the current study available from the corresponding author on reasonable request.
